# Manipulating the banana rhizosphere microbiome for biological control of Panama disease

**DOI:** 10.1038/srep11124

**Published:** 2015-08-05

**Authors:** Chao Xue, C. Ryan Penton, Zongzhuan Shen, Ruifu Zhang, Qiwei Huang, Rong Li, Yunze Ruan, Qirong Shen

**Affiliations:** 1 Jiangsu Collaborative Innovation Center for Solid Organic Waste Utilization and National Engineering Research Center for Organic-based Fertilizers, Department of Plant Nutrition, Nanjing Agricultural University, Nanjing, 210095, PR China; 2 College of Letters and Sciences, Faculty of Science and Mathematics, Arizona State University, Mesa, AZ, 85212, USA; 3 Hainan Key Lab for Banana Planting, College of Agriculture, Hainan University, Haikou, Hainan, 570228, PR China

## Abstract

Panama disease caused by *Fusarium oxysporum* f. sp. *cubense* infection on banana is devastating banana plantations worldwide. Biological control has been proposed to suppress Panama disease, though the stability and survival of bio-control microorganisms in field setting is largely unknown. In order to develop a bio-control strategy for this disease, 16S rRNA gene sequencing was used to assess the microbial community of a disease-suppressive soil. *Bacillus* was identified as the dominant bacterial group in the suppressive soil. For this reason, *B. amyloliquefaciens* NJN-6 isolated from the suppressive soil was selected as a potential bio-control agent. A bioorganic fertilizer (BIO), formulated by combining this isolate with compost, was applied in nursery pots to assess the bio-control of Panama disease. Results showed that BIO significantly decreased disease incidence by 68.5%, resulting in a doubled yield. Moreover, bacterial community structure was significantly correlated to disease incidence and yield and *Bacillus* colonization was negatively correlated with pathogen abundance and disease incidence, but positively correlated to yield. In total, the application of BIO altered the rhizo-bacterial community by establishing beneficial strains that dominated the microbial community and decreased pathogen colonization in the banana rhizosphere, which plays an important role in the management of Panama disease.

Banana (*Musa* spp.) is a widely cultivated cash crop in tropical and subtropical climatic regions. This disease, caused by the infection of the soil-borne hyphomycete *Fusarium oxysporum* f. sp. *cubense* (FOC), leads to banana rot and death and is known as vascular fusarium-wilt or Panama disease^1^. Once infected, it is almost impossible to remove the pathogen from the soil. In the 1950s, Panama disease devastated the dominant banana cultivar Gros Michel, forcing producers to switch to Cavendish, which was originally resistant to FOC^2^. As such, Cavendish became the most popular banana cultivar, accounting for nearly all of the global banana export trade^3^. However, a new variant of FOC (TR4) has been found to have a particularly devastating effect on the Cavendish cultivar^4^. This new variant, which was originally limited to parts of Asia (Indonesia, Philippines, and China) and Australia, has recently been discovered in Latin America, raising fears of another crop collapse^5^,^6^.

Panama disease is conventionally suppressed by soil sterilization through fumigation (methyl bromide) and fungicide application^7^,^8^. However, these techniques can cause environmental contamination, as many soil-sterilizing chemicals are toxic and banned^9^,^10^. Creating new banana cultivars, which are completely resistant to FOC-TR4 by traditional breeding, has been impeded by triploidy. Further progress by genetic engineering has thus far been limited^11^. Biological control, the use of living organisms to manage infected soils, has the potential to avoid these issues and while simultaneously improving soil quality^12^.

A disease suppressive soil is described as soil present in a severely disease infested field that has the potential to decrease pathogenesis^13^. Previous studies from diverse soils have attributed this suppression to the composition of the soil microbial community^14^,^15^. Microorganisms beneficial to disease suppression are commonly isolated, cultured, and applied to soil for potential disease control^16^,^17^. However, their survival, stability, and influence on the nascent microbial community are largely unknown, resulting in an inconsistent field performance. Compost is widely applied in agricultural production to improve both soil and product quality and can be specifically produced to suppress plant disease^18^. A possible suppression mechanism is the presence of multiple beneficial strains such as *Trichoderma*, *Actinobacteria*, and *Bacillus*. These have been identified as dominant microbial groups in compost, serving to inhibit pathogen growth and confer plant protection from pathogen infection^19^,^20^. However, due to high temperature, many of these beneficial microorganisms form spores during compost production and thus require time to germinate after application. Research has also shown that compost had no effect on the suppression of fusarium-wilt, with a marked increase in pathogen abundance^21^. Together, these results suggest that the use of compost to control plant disease is neither stable nor suppressive of pathogen incidence. However, we have developed an optimized biological strategy, which results in consistent disease suppression to field-based soil-borne disease on diverse crops^22^,^23^,^24^,^25^,^26^. This was accomplished by nursery pot application of a bioorganic fertilizer (BIO) composed of a fermentation product derived from a beneficial bacterial strain mixed with an amino acid fertilizer (Fig. 1).

In this study, we describe how a formulated bioorganic fertilizer specifically suppresses banana Panama disease. Comparisons of microbial community structure between disease-suppressive and diseased soils, beneficial strain isolation, and a field study implementing BIO application are investigated. We also demonstrate that BIO application establishes a beneficial taxa-dominated microbiome in the banana rhizosphere, which plays an important role in the management of *Fusarium* wilt disease in banana plantations. The following specific questions were addressed to further support our concept of manipulating the rhizosphere microbiome in order to suppress soil-borne disease: (i) Is the health status of the banana plant associated with the composition of the soil microbial community? (ii) What is the microbial community in disease-suppressive soil? (iii) Can BIO suppress Panama disease by manipulating the rhizosphere microbial community?

## Results

### Disease suppressive soil microbiome and isolation

Microbial community differences between the disease-suppressive and diseased soils were estimated by comparing 16S rRNA gene clone libraries. In total, 200 sequences were obtained for analysis (100 per sample). Clustering at 3% nucleotide dissimilarity resulted in 180 OTUs that were classified into 7 phylum, 16 classes, and 87 genera. *Bacillus* was the most dominant bacterial group in the disease suppressive soil followed by *Rhizobium, Bhargavaea, Pseudolabrys*, and *Sinorhizobium*. In comparison, the diseased soil harbored *Acidovorax, Filimonas, Methylophilus, Oxalicibacterium*, and *Sphingomonas* among the top 5 abundant genera (Fig. S2).

In total, 100 isolates were obtained from the disease-suppressive soil samples. These were tested by inoculation against *Fusarium oxysporum* f. sp. *cubense* (FOC) on potato dextrose agar plates. The isolate (NJN-6) with the highest suppression towards FOC was selected and identified as a member of the *Bacillus amyloliquefaciens* group (GenBank accession number GQ452909, CGMCC accession number 3183), based on a maximum likelihood *16S rRNA* based phylogenetic tree (Fig. 2).

### Field experiment

Liquid fermentations of strain *B. amyloliquefaciens* NJN-6 were blended with pig manure compost and amino acid fertilizer to produce the specific bioorganic fertilizer (BIO). This was applied to suppress Panama disease in a field with a 65% banana death rate during the last growing season. Overall, BIO succeeded in controlling Panama disease with a promotion of banana yield (Fig. S1). For the control treatment (CK), wilt disease was extremely severe with an average incidence rate of 45% for all CK plots, average unit yields of 29 kg/plant, and plot yields of 482 kg/plot (Fig. 3). After BIO application, the average disease incidence significantly (*t*-test, *p* < 0.05) decreased to 14%, resulting in a biological control percentage of 68.5% and a significantly increased unit yield (36 kg/plant) (*t*-test, *p < *0.05) and plot yield (927 kg/plot) (*t*-test, *p < *0.05)) (Fig. 3).

### Pathogen quantification

The number of *F. oxysporum* f. sp. *cubense* (FOC) colony forming units (CFU) in the banana rhizosphere was quantified by plating soil series dilutions on Komada’s selective medium K2 Petri plates for all samples collected in 2010. The FOC colonization was significantly lower in the disease-suppressive, compared to diseased soils (ANOVA, *p < *0.05) (Fig. 4). All diseased samples in both treatments contained significantly higher rhizosphere FOC abundances (ANOVA, *p < *0.05). Pearson correlations revealed that FOC colonization was highly correlated to plant disease incidence (*r* = 0.73, *p < *0.05), unit yield (*r* = −0.92, *p < *0.05) and plot yield (*r* = −0.85, *p < *0.05).

### Pyrosequencing data

After quality trimming, the number of sequences obtained using pyrosequencing ranged from 7087 to 11727 per sample. Rarefaction curves were not saturated in all cases (Fig. S3). After re-sampling, the average number of OTUs per sample was 2482 ± 349. All reads were classified using the RDP naïve Bayesian Classifier into 28 phyla, 62 classes and 685 genera with unclassified reads ranging from 1.8% to 13.8% at the phylum level, 5.2% to 17.5% at the class level and 30.6% to 54.4% at the genus level.

#### Effect of treatment and health status on bacterial community composition

Ordinations illustrated that all samples could be separated according to both health status and treatment (Fig. 5). PERMANOVA showed significant differences according to treatment (F = 2.66, *p* < 0.05), health status (F = 1.60, *p < *0.05) and the treatment × health status interaction term (F = 1.67, *p* < 0.05). Overall sample dispersion was not significant (PERMDISP; *p* > 0.05). Multiple regression tree analyses resulted in a primary division of the bacterial community according to treatment, followed by health status (Fig. 6). *Acidobacteria-Gp6* contributed most to the community differences between the healthy soil after BIO application and diseased soil in the control treatment, (SIMPER) followed by *Gemmatimonas, Ohtaekwangia, Acidobacteria-*Gp1*, Steroidobacter, Bacillus, Streptophyta, Flavobacterium*, and *Dongia* (Table S2). Overall bacterial community composition was significantly correlated to disease incidence (Mantel; *r = *0.51, *p* < 0.05), banana unit yield (*r = *0.60, *p < *0.05) and plot yield (*r* = 0.54, *p < *0.05).

#### Effect of bioorganic fertilizer on bacterial communities

After BIO application (BIOH), healthy samples shared more OTUs with the disease suppressive soil (CKH) than diseased samples in the control treatment (CKD), and more shared OTUs were classified as Bacillus. In total, 48 OTUs were shared by BIOH and CKH samples (reads: BIOH/CKH = 7.67%/9.96%), 40 OTUs were shared by all the CKD and CKH samples (reads: CKD/CKH = 7.93%/8.85%), while 83 shared OTUs (reads: BIOH/CKD = 8.43%/11.89%) were observed in all the BIOH and CKD samples. The most abundant bacterial taxa of BIOH and CKH were *Bacillus* (reads: CKH/BIOH = 1.19%/3.02%). Only one shared-OTU, which was classified as *Bacillus*, was observed in BIOH and CKD (reads: BIOH/CKD = 0.21%/0.21%), and in CKD and CKH (reads: CKD/CKH = 0.21%/2.13%). Pearson correlations revealed that *Bacillus* colonization was positively correlated to banana unit yield (*r* = 0.82, *p < *0.05) and plot yield (*r* = 0.81, *p < *0.05), but negatively correlated to disease incidence (*r* = −0.80, *p < *0.05) and FOC abundance in banana rhizosphere (*r* = −0.57, *p < *0.05).

#### Disease suppressive microbiome

Bacterial community structure in the disease-suppressive (CKH) and diseased (CKD) soils within the control treatment was compared to elucidate the nature of the banana rhizosphere disease-suppressive microbiome. The microbial communities of CKH and CKD differed from the phylum level to genus level. At the phylum level *Firmicutes* and *Actinobacteria* were more abundant in CKH, while *Verrucomicrobia*, *Bacteroidetes*, *Proteobacteria*, and *Gemmatimonadetes* were more abundant in CKD (*t*-test, *p < *0.05). *Bacillus* was the top contributor (SIMPER; 5.5% of the total Bray-Curtis distance) to the community differences between CKH and CKD, followed by *Acidobacteria* GP6, *Gemmatimonas, Flavobacterium, Acidobacteria* GP1, *Rhodanobacter, Ohtaekwangia, Sphingobium, Steroidobacter*, and *Burkholderia* (Table S1).

In total, only 121 OTUs (21.44% of reads) were shared by all disease-suppressive samples (CKH), of which 56 OTUs (6.45% of reads) were classified as *Bacillus*, while only one OTU (0.21% of reads) of 208 shared by all CKD samples was classified as *Bacillus*. This ‘core microbiome’ from the disease-suppressive soil was dominated by *Bacillaceae*, followed by *Hyphomicrobiaceae*, *Gaiellaceae*, *Bradyrhizobiaceae*, *Sphingomonadaceae*, *Rhodospirillaceae*, *Paenibacillaceae*, *Nitrospiraceae*, and *Streptomycetaceae*.

## Discussion

An understanding of the composition of the rhizosphere microbiota in relation to plant health is essential for the prevention and treatment of plant diseases. In this context, we compared the rhizosphere bacterial community compositions of healthy plants with plants infected by Panama disease. Banana plant health status was associated with the composition of the rhizo-bacterial community in each treatment as revealed by beta-diversity comparisons and mantel tests. Two mechanisms may be responsible for these changes. First, plant health status may affect root exudates, which in turn have a selective effect on the rhizosphere bacterial community^27^,^28^. Second, *Fusarium oxysporus* f. sp. *cubense* (FOC) infection resulted in larger populations of FOC colonizing the rhizosphere that, through direct agonistic/antagonistic interactions, may serve to alter the composition of the rhizosphere community. However, it should be noted that pathogenic or non-pathogenic FOC could not be separately quantified^29^, so the larger FOC populations may include a non-pathogenic component. However, field performance indicated that the non-pathogenic FOC were not a major component of FOC detected in the diseased banana rhizosphere.

As a first step to disease management, the microbial community composition of a native disease-suppressive soil with Panama severe disease was assessed with clone libraries and pyrosequencing in order to identify key microbial taxa associated with suppression. Clone sequencing and pyrosequencing results agreed, with a high relative abundance of *Bacillus* in the healthy soil and a negative correlation to FOC colonization in banana rhizosphere. *Bacillus* is a well-known plant growth promoting (PGPR) rhizo-bacterial group that has been shown to produce antifungal compounds and phytohormones in disease-suppressive soils^30^. Second year pyrosequencing data also showed *Bacillus* comprised one-third of the “core microbiome” in disease-suppressive samples. This is in contrast to a previous study on the microbial community of potato common scab-suppressive soil in which *Bacillus* was more abundant in disease-conducive soil^31^. This illustrates contrasting roles of different members of the soil community according to crop and other environmental influences. As such, it is important to first determine the composition of the disease suppressive soil microbial community before the development of biocontrol products. It should also be noted that the shorter pyrosequencing read fragments could not distinguish *B. amyloliquefaciens* from other *Bacillus* species with up to only 54.4% of sequences classified at the genus level at 50% confidence.

Other potentially beneficial groups were associated with disease suppression such as *Burkholderia, Gaiellaceae, Paenibacillaceae, and Streptomycetaceae*. Both *Gaiellaceae* and *Streptomycetaceae* belong to *Actinobacteria*, reported to be a dominant group in soils resistant to fusarium-wilt of banana^32^. Pan *et al*.^33^ found that *Burkholderia sp*. could colonize the surface of FOC hyphae and that the fungal macrospores resulted in mycelial deformation with terminal and intercalary swelling. *Paenibacillaceae sp*. has also been found to produce fusaricidins, an antifungal compounds group that suppresses *F. oxysporum* f. sp*. nevium*^34^. In this study, the combination of clone library data and culturing led to the selection of *B. amyloliquefaciens* NJN-6 as a potential biocontrol agent against Panama disease. Gram positive, spore-producing *Bacillus* strains are ideal for commercial production and mixing in organic fertilizers and soil, due to their survival characteristics^35^.

Previous research has shown that compost application had no effect on *Fusarium* wilt suppression and that pathogen abundances actually increased^21^. This led us to utilize nursery pot applications of bioorganic fertilizer (BIO) produced by blending of a *B. amyloliquefaciens* NJN-6 fermentation with an amino acid fertilizer and compost. In this study, BIO was expected to complement the establishment of a beneficial strain-dominated microbial community in the banana rhizosphere. Although there was a significant difference in overall microbial community structure between healthy and diseased samples, the rhizo-bacterial community was most affected by the addition of BIO. Multiple regression tree analyses confirmed that the BIO influence on the microbiome was able to overcome the effect of plant disease status. As a source of nutrition for bacteria involved in biocontrol, organic fertilizer may aid in antagonistic colonization in the banana rhizosphere by increasing soil microbial biomass and activity^36^,^37^, resulting in a doubling of crop yield coupled with 68.5% biocontrol efficacy of banana Fusarium wilt. This result was agreed with other studies involving *Cucumis melo* melon^23^, tobacco^38^, cucumber^39^ and watermelon^40^.

The seedlings used in this study were generated through tissue culture and the germ-free seedling roots first came into contact with BIO during the nursery pot period, followed by the FOC infected field soil after transplantation. However, the field soil was also supplemented with BIO before the transfer of banana seedlings from the pot to the field. This likely resulted in an enhanced colonization of beneficial bacteria that contributed to the persistent beneficial effects^22^. This initial colonization by beneficial bacteria may also initially induce banana seedling systemic disease resistance^41^. The application of the *B. amyloliquefaciens* NJN-6 dominated BIO appeared to initiate microbial community shifts in the banana rhizosphere, leading to decreased FOC abundance, similar to results found in relation to watermelon^42^ and cucumber^43^. In addition to systemic resistance, direct antagonism to the pathogen through nutrient or niche competition or by antifungal production may be involved. For example, Ling *et al*.^44^ reported that biocontrol agents affected watermelon root exudates, leading to the decrease in the conidial germination of *Fusarium oxysporum* f. sp. *niveum*.

The suppression of Panama disease by *B*. *amyloliquefaciens* colonization of the banana rhizosphere may be due to plant growth promotion. Specifically, strain NJN-6 has been found to produce indole 3-acetic acid (IAA) that enhances root system development, leading to increased root exudate production and a larger rhizosphere habitat^45^. Two types of antifungal lipopeptides (iturin and bacillomycin D) and 18 volatile antifungal compounds that suppress FOC have also been identified^46^. Ultimately, a *Bacillus* dominated rhizosphere bacteria community was established that acted through direct and/or indirect antagonism against the pathogen, resulting in increased crop yields and decreased disease incidence.

## Conclusion

In this study, we described the development of a specific Panama disease suppressive bioorganic fertilizer (BIO) to control plant disease and promote plant growth. In addition, comparisons of disease suppressive and diseased soil microbial communities, screening of biocontrol agents, and nursery pot applications of the bio-product were investigated. Both clone and pyrosequencing data showed that *Bacillus* was the most abundant bacterial group in disease suppressive soils in the banana rhizosphere and that plant health status was correlated to soil microbial community composition. Field results demonstrated that the practical application of the BIO is a promising strategy for disease control and growth promotion in banana production. In addition, BIO application altered the rhizo-bacterial community by establishing beneficial strains that dominated the microbial community and decreased pathogen colonization pathogen in the banana rhizosphere, which plays an important role in the management of *Fusarium* wilt disease in banana plantations.

## Materials and Methods

### Field site description

The banana plantation under study is located in Ledong county (E 109°17’, N 18°73’), Hainan province, China, where Panama disease is widespread and has caused severe crop losses. The study site was a flat banana field that suffered severe Panama disease from 2007–09 and had been under continuous banana cropping for 5 years since 2005. In 2009, 65% of the banana plants died in this field. The germ-free banana (*Musa* AAA Cavendish cv. Brazil) seedlings used in this study were the same as previously planted in this field, supplied by Hainan Wan Zhong Co., Ltd., Hainan, China.

### Sampling, DNA extraction, and 16S rRNA gene library construction

Rhizosphere soil samples were collected after harvest in 2009. Three replicates of individual disease-suppressive banana (no disease symptom observed) roots were collected in this field as well as the individual wilted banana (infected banana plants) roots. Soil remaining on the root segment after a strongly shaking was considered to be rhizosphere soil^47^. Ten grams of fresh root was added to 90 ml sterilized water followed by 10 min ultra sonication and 30 min shaking at 200 rpm. Part of this soil suspension was used for FOC quantification. Rhizosphere soils from this suspension were collected by centrifugation at 12,000 × g for 15 min at 25 °C. DNA extraction using the Power soil DNA Extraction kit (MOBIO Laboratories, Carlsbad, CA, USA) was conducted following the manufacturer’s instructions. For DNA extraction, all the suppressive and diseased replicated samples were combined to make one sample per treatment.

The primer set 27f and 1492r was used to amplify the 16S rRNA genes^48^. For each sample, 5 replicated 25 μl PCRs were performed, with each mixture containing 20 ng of genomic DNA, 2.5 μl of Mg^2+^ free 10 × PCR buffer, 2 μl of Mg^2+^ (15 mM), 2 μl of mixed dNTPs (2.5 mM each), 10 pmol each forward and reverse primer, and 2 U of Taq polymerase (5 U/μl, TaKaRa, Dalian, China). Amplification was performed with an initial denaturation of 4 min at 95 °C; followed by 30 cycles of 1 min at 94 °C, 1 min at 55 °C and 1.5 min at 72 °C, and a final extension at 72 °C for 10 min. The PCR products were purified using the Axygen PCR clean-up kit, ligated into a pMD19-T Vector (Takara), and transformed into *E. coli* DH5α. Transformants were randomly selected by plating onto Luria-Bertani /Ampicillin agar plates with IPTG and X-Gal and incubated at 37 °C overnight. White clones from each soil samples were randomly picked and sequenced using an ABI 3730 gene analyzer.

### Bio-control agent isolation

Disease suppressive banana roots with rhizosphere soil from a severe Panama disease field were collected and washed with sterilized water and a soil suspension dilution series was plated on 30% beef extract medium (0.9 g beef extract, 3 g Peptone, and 2 g NaCl in 1 L water). Single colonies were obtained and cultured with *F. oxysporum* f. sp. *cubense* race 4 (ACCC accession number 38875, Agricultural Culture Collection of China) on potato dextrose agar plates to observe whether the strains inhibited pathogen growth. The isolate, which showed extremely strong suppressive efficacy on FOC growth, was selected and identified using 16S rRNA gene sequencing. A maximum composite likelihood phylogenetic tree of the 16S rRNA sequence was generated by MEGA 5.

### Bioorganic fertilizer preparation

The banana specific bioorganic fertilizer (BIO) used in this study was a mixture of organic fertilizer and the antagonistic isolates described above at approximately 1 × 10^8^ CFU g^−1^ fresh weight. The organic fertilizer was a mixture of amino acid fertilizer and pig manure compost (1:1, w/w) as described previously^49^. Amino acid fertilizer contained a rapeseed oil extraction residue, which was enzymatically hydrolyzed by aerobic microbial fermentation at <50 °C for 7 days. Pig manure compost was fermented using pig manure at temperatures ranging from 30 to 70 °C for 25 days (Tianniang Ltd., Suzhou, China). The BIO was stored at <25 °C for 7 days before use.

### Field experimental design

**Figure 1:**
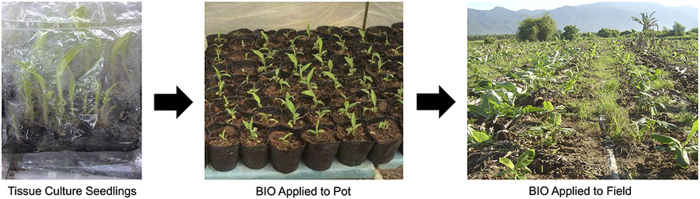
Roadmap of nursery pot application of bioorganic fertilizer (BIO). Picture taken by first author Chao Xue.

**Figure 2:**
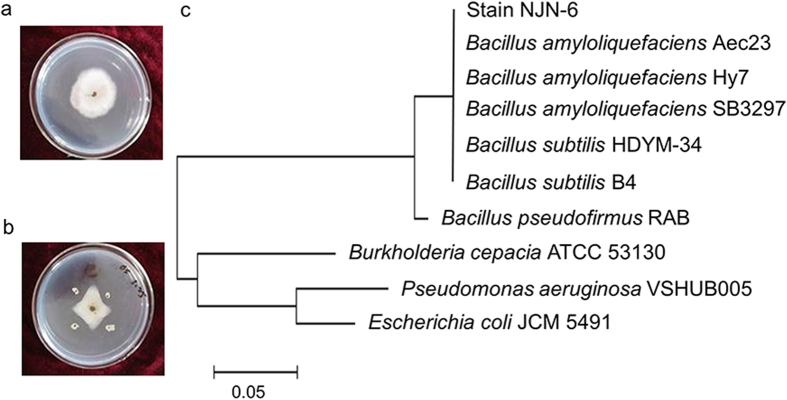
*In-vitro* antagonism against *Fusarium oxysporum* f. sp. cubense race 4 (FOC) by strain NJN-6. **a**) Control, FOC inoculated with sterilized water; **b**) antagonism against FOC by NJN-6; **c**) maximum composite likelihood phylogenetic tree of strain NJN-6.

**Figure 3:**
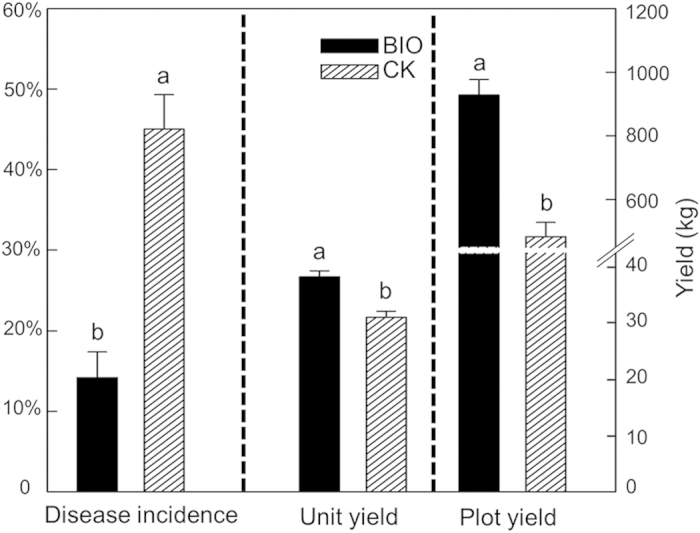
Yield and disease incidence of the bioorganic fertilizer (BIO) and control (CK) treatments. The significance of the difference was determined by two-way unpaired *t*-tests. Bars sharing the same character represent the lack of a significant difference (*p > *0.05).

**Figure 4:**
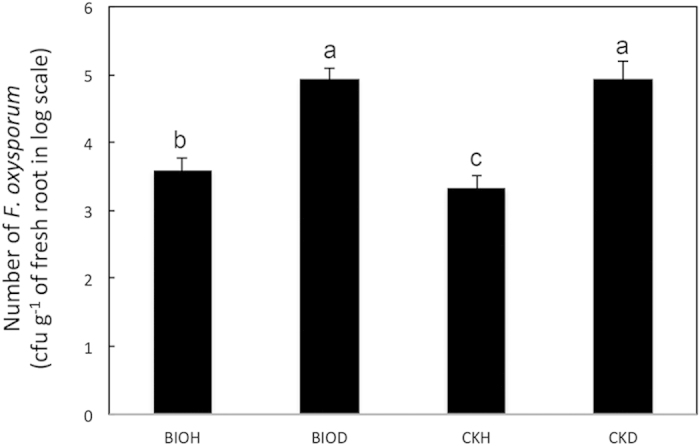
Number of *Fusarium oxysporus* f. sp. *cubense* (FOC) colony forming units (CFU) of disease-suppressive (CKH) and diseased (CKD) samples in the control treatment and healthy (BIOH) and diseased (BIOD) samples within the bioorganic fertilizer treatment. The significance of the difference was determined by one-way ANOVA (n = 4). Bars shared the same character represent a lack of significant difference (*p > *0.05).

**Figure 5:**
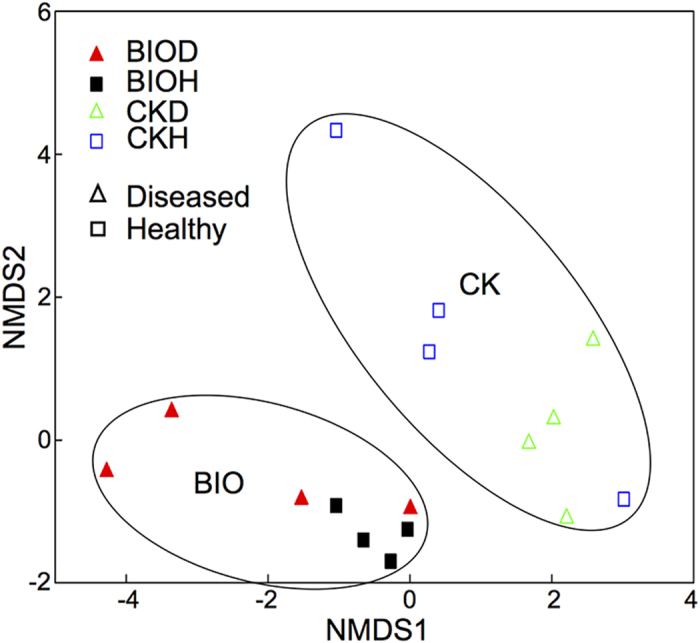
Non-metric multidimensional scaling analysis based on Bray-Curtis dissimilarity between all sample sets. CKH and CKD are healthy and diseased samples collected in control treatment, respectively. BIOH and BIOD are healthy and diseased samples collected in bioorganic fertilizer treatment, respectively. BIO and CK are samples collected in bioorganic fertilizer and control treatments, respectively, regardless of plant health status.

**Figure 6:**
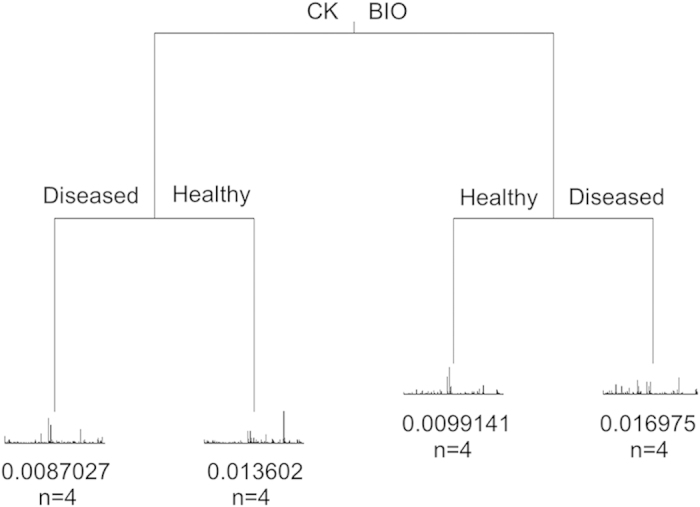
Multiple regression tree (MRT) analysis of plant health status and treatment effects on microbial composition. BIO and CK represent bioorganic fertilizer and control treatments, respectively.

After the 2009 harvest, the field was divided into 8 plots with 2 treatments; one was planted with BIO and the other was planted with organic fertilizer, which served as a control. Each treatment had 4 parallel plots randomly assigned in 4 of the 8 plots (166 m^2^/plot). In the BIO treatment, banana seedlings were grown in nursery cups for 45 days and then pots for 60 days supplemented with BIO (4% per cup and pot, w/w) that were then transplanted into the BIO supplemented holes (500 g /hole) in the field plots when the plants had 5 to 6 true leaves (Fig. 1). The control treatment was conducted in an identical fashion using the organic fertilizer prepared as described above to replace BIO. The disease incidence (DI) and the yield were calculated immediately after harvest (~ 8 months after field transplantation). DI was evaluated by plot as the percentage of infected plants divided by the total number of plants. The biological control percentage was obtained according to the equation: S = (A − B)/A, where A is DI of the control treatment and B is DI of the BIO treatment. Unit yields were assessed as a mean cumulative fresh weight of 5 banana plants. Plot yields were calculated by unit yield timed number of plants survived in this plot.

### Sampling, DNA extraction, and 16S rRNA gene pyrosequencing

Rhizosphere soil samples were collected after the 2010 harvest. The FOC-infected banana roots were collected in the control treatment (CK) and bioorganic fertilizer treatment (BIO) and assigned as CKD and BIOD, respectively. Healthy banana roots were collected in BIO and CK and assigned as BIOH and CKH, respectively. Five individual plants were randomly collected in each plot and mixed as one sample. Each sample had 4 replicates collected from the 4 parallel plots. Root soil was collected and DNA was isolated as described above. Replicated soil samples were not combined, but kept as 4 replicates.

The 454 pyrosequencing fusion primer set that included the 454 sequencing adaptor, barcode and the universal 16S rRNA gene primer set (27f/533r)^50^ was used to amplify the 16S rRNA genes of the 2010 samples. For each sample, 3 replicates of 20 μl PCR reactions were performed, with each mixture containing 2 μl of soil DNA, 2 μl of 10 × PCR buffer with 18 mM MgCl_2_, 0.2 μl BSA (10 mg/ml, Promega), 0.75 μl of mixed dNTPs (10 mM, Invitrogen), 10 pmol each forward and reverse primer, and 2.5 U of high fidelity Taq polymerase (5 U/μl, Roche). Amplification was performed with an initial denaturation of 3 min at 95 °C followed by 30 cycles of 45 s at 94 °C, 45 s at 59 °C and 1 min at 72 °C, and a final extension of 7 min at 72 °C. The PCR products were purified using the Axygen DNA gel recovery kit (Axygen Bio, Union City, CA, USA). Sequencing was performed by Majorbio Biotech Co., Ltd. (Shanghai, China).

### Pathogen quantification

This soil solution obtained from root wash was used for pathogen quantification for all the samples collected in 2010. The number of *F. oxysporum* f. sp. *cubense* (FOC) colony forming units (CFU) colonizing the banana rhizosphere was quantified by plating soil series dilution solutions from rhizosphere soil suspensions on petri plates with modified Komada’s selective medium, as described previously^51^. The basal medium contains the following in 900 ml distilled water: K_2_HPO_4_, 1 g, MgSO_4_•7H_2_O, 0.5 g, KCl, 0.5 g, Fe-Na-EDTA, 0.01 g, D-Galactose, 10 g (in contrast to 20 g in Komada’s original medium), L-Asparagine, 2 g, and 16 g of agar. The basal medium was mixed with 100 ml of solution containing the following agents: 0.9 g PCNB (pentachloronitrobenzene, 75% WP), 0.5 g Na_2_B_4_O_7_•10H_2_O, 0.45 g oxgall, and 0.3 g streptomycin sulfate. The pH was adjusted to 3.8 ± 0.2 with 10% phosphoric acid. Plates were stored at 25 °C after plating for 10 days before colony counting. Triplicates were conducted for each sample.

### Data processing

Clone library sequences were analyzed with Mothur^52^, with chimera filtering before alignment and classification. Pyrosequencing data were barcode sorted and processed using the RDP processing pipeline with the removal of low quality (Q score < 20) and short reads (length < 200)^53^. Chimeric reads were removed using Usearch chimera check in *de novo* mode. Samples were randomly subsampled to 7018 reads per sample. For both the clone library and pyrosequencing data, sequences were aligned and clustered at 97% nucleotide identity and taxonomy was assigned using RDP Naive Bayesian classifier^54^.

For pyrosequencing data, non-metric multidimensional scaling (NMDS) was performed to illustrate beta-diversity between individual samples (Bray-Curtis distances between samples) after a hellinger transformation of the raw data. Similarity percentage (SIMPER) analysis was conducted to elucidate the indicator genera to the overall Bray-Curtis distances between samples^55^. Core OTUs were computed using a python script from QIIME^56^. Multiple regression tree (MRT) analysis was conducted to evaluate the treatment and plant health status effects on the whole soil microbial community^57^. Permutational multivariate analysis of variance (PERMANOVA) was performed to evaluate the significant differences of microbial community composition between sample sets^58^. Permutational analysis of multivariate dispersions (PERMDISP) was conducted to evaluate the significant differences in replicate dispersion among samples^59^. Mantel tests were used to identify correlations between microbial groups and disease incidence and yield^60^. Correlations between individual microbial groups and disease incidence and yield were conducted by Pearson correlation and TDIST tests for *r* and *P* values. To determine the significance of the differences, two-tailed, unpaired t–tests were performed on individual microbial groups in SIMPER analysis, disease incidence, unit yield, and plot yield. ANOVA followed by the LSD test in SAS (version 6.1) (SAS Inc., Cary, NC, USA) was performed on FOC quantification data.

All clone library sequences were deposited at GenBank (Accession numbers: KJ600797 - KJ600996). The pyrosequencing sequences were deposited in the NCBI Sequence Read Archive (SRA) database (Accession number: SRR1185515- SRR1185517).

## Additional Information

**How to cite this article**: Xue, C. *et al*. Manipulating the banana rhizosphere microbiome for biological control of Panama disease. *Sci. Rep*. **5**, 11124; doi: 10.1038/srep11124 (2015).

## Supplementary Material

Supplementary Information
