# Corrigendum: Manipulating the banana rhizosphere microbiome for biological control of Panama disease

**DOI:** 10.1038/srep14596

**Published:** 2015-10-13

**Authors:** Chao Xue, C. Ryan Penton, Zongzhuan Shen, Ruifu Zhang, Qiwei Huang, Rong Li, Yunze Ruan, Qirong Shen

Scientific Reports 5: Article number: 1112410.1038/srep11124; published online: 08052015; updated: 10132015

The Introduction of this Article incorrectly cites reference 6 as:

Kema, G. H. J. & Weise, S. Pathogens: Appeal for funds to fight banana blight. *Nature* **504**, 218 (2013).

The correct reference 6 appears below as reference 1.

Butler, B. Fungus threatens top banana. *Nature*. **504**, 195–196 (2013).

As a result,

“This new variant, which was originally limited to parts of Asia (Indonesia, Philippines, and China) and Australia, has recently been discovered in Latin America, raising fears of another crop collapse^5,6^.”

should read:

“This new variant, which was originally limited to parts of Asia (Indonesia, Philippines, and China) and Australia, has recently been discovered in Africa and Mid-East, raising fears of another crop collapse^5^^,^^6^.”

