# Supplementary material for: Manipulating the banana rhizosphere microbiome for biological control of Panama disease
Source: Sci Rep. 2015 Aug 5;5:11124. doi: 10.1038/srep11124 (PMC4525139; doi:10.1038/srep11124)
Supplement: Supplementary Information [file srep11124-s1.pdf]

Manipulating the banana rhizosphere microbiome for biological control of Panama disease

Chao Xue[a], C. Ryan Penton[b], Zongzhuan Shen[a], Ruifu Zhang[a], Qiwei Huang[a], Rong Li[a], Yunze Ruan[c], Qirong Shen[a, †]

[a] Jiangsu Collaborative Innovation Center for Solid Organic Waste Utilization and National Engineering Research Center for Organic-based Fertilizers, Department of Plant Nutrition, Nanjing Agricultural University, Nanjing, 210095, PR China

[b] College of Letters and Sciences, Faculty of Science and Mathematics, Arizona State University, Mesa, AZ, 85212, USA

[c] Hainan Key Lab for Banana Planting, College of Agricultural, Hainan University, Haikou, Hainan, 570228, PR China

[†] Corresponding author. Tel: +86 025 84395366; Fax: +86 025 84395212; E-mail:shenqirong@njau.edu.cn

Supplementary figures

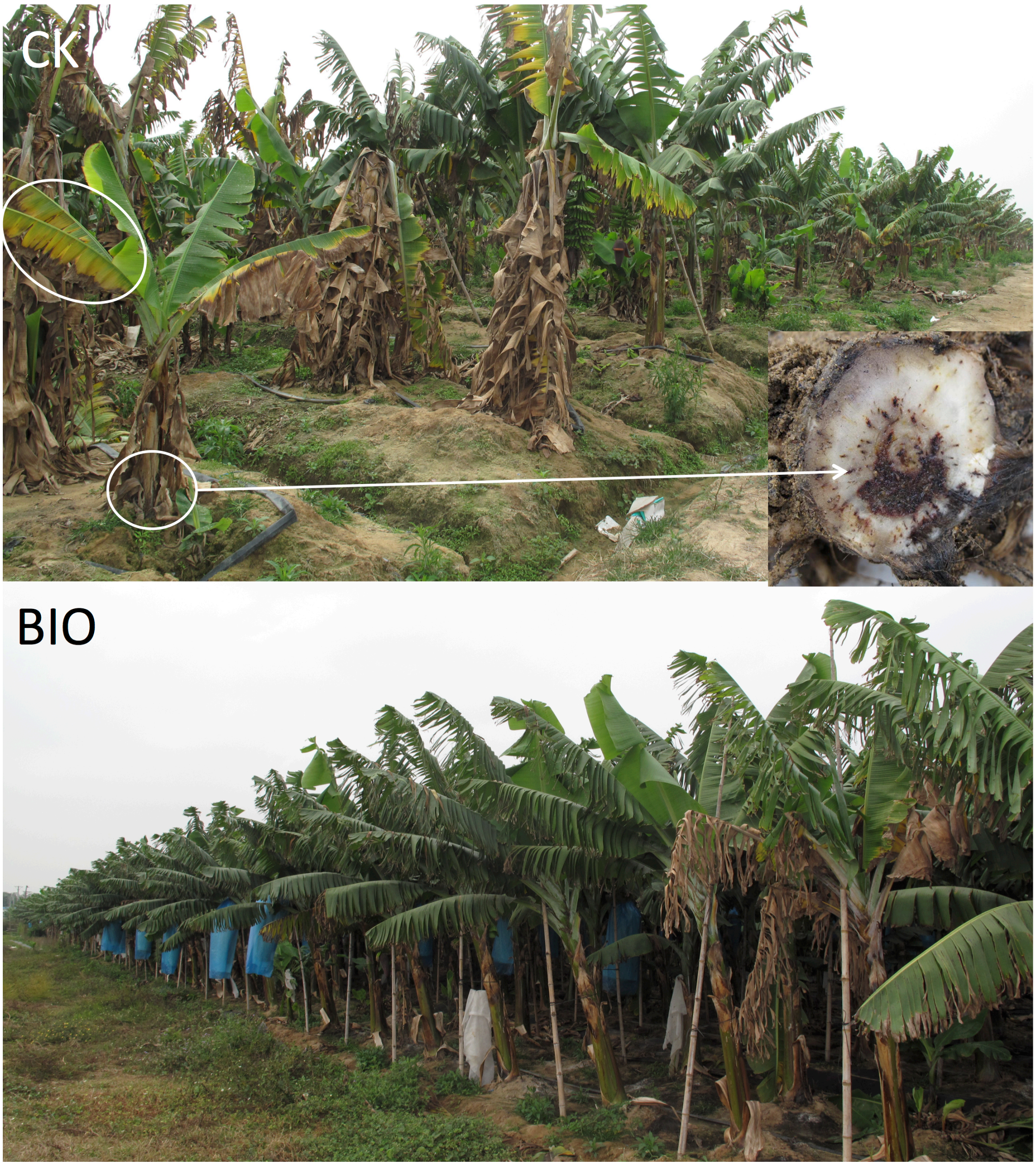

Fig. S1 Field experiment with bioorganic fertilizer (BIO) and control (CK) treatment. Typical symptoms of Panama disease with wilted leaves and pathogen colonization at the bottom of the stem. Picture taken by first author Chao Xue

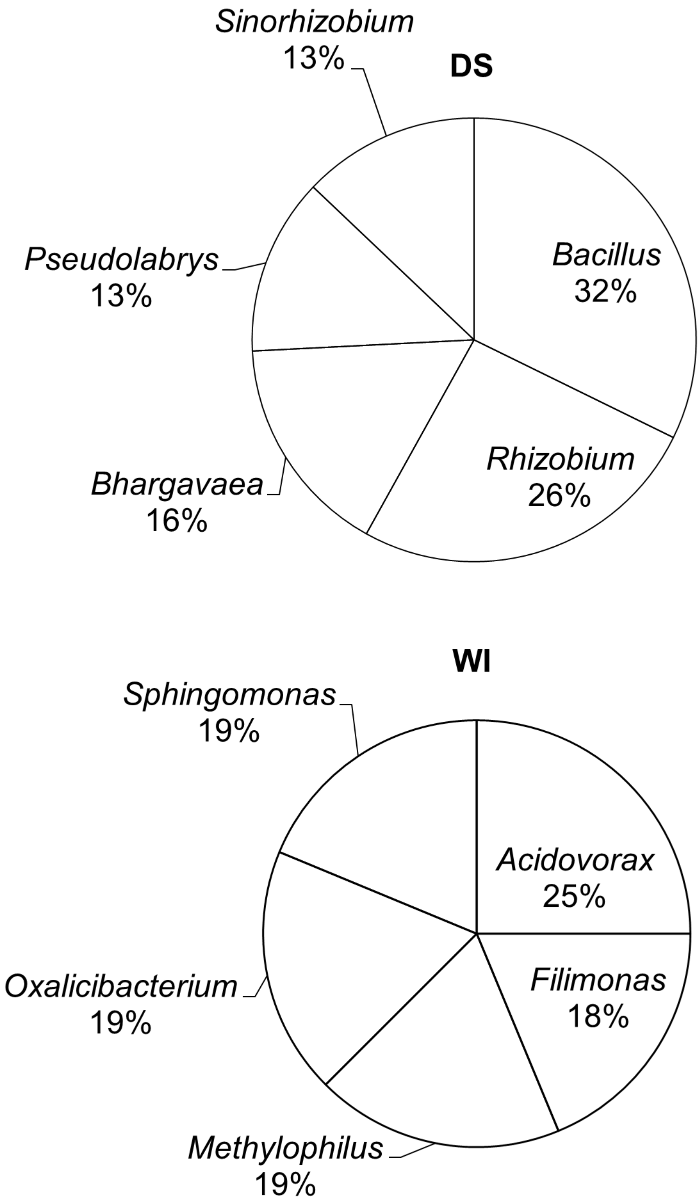

Fig. S2 Top five abundant genera in disease suppressive and diseased soil samples

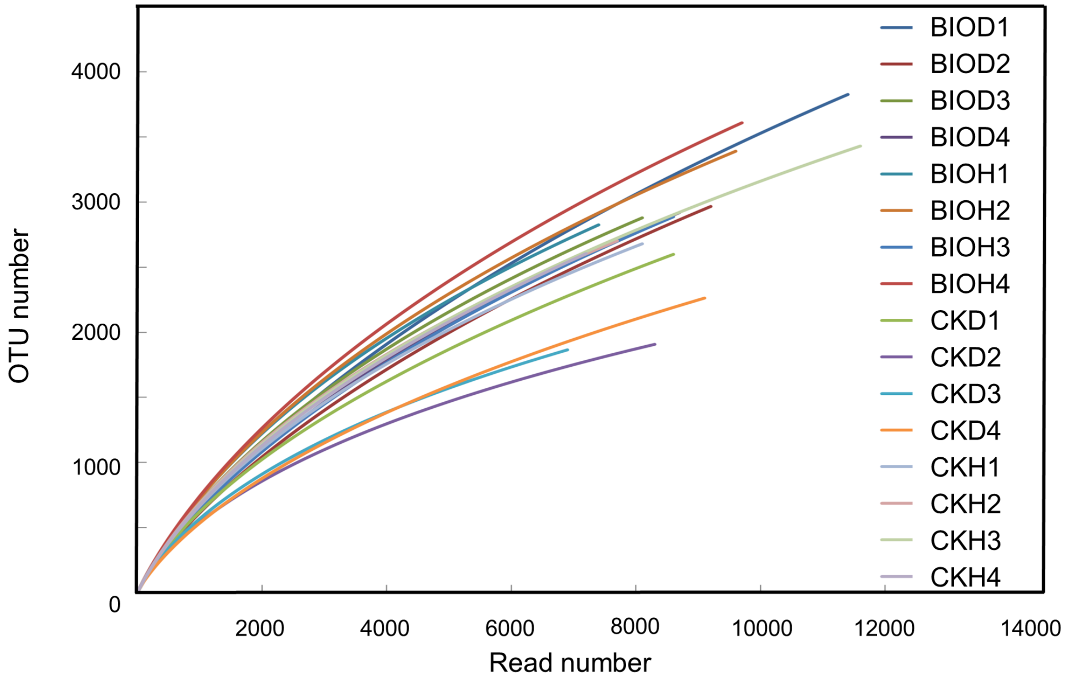

Fig. S3 Rarefaction curves of all samples obtained from pyrosequencing data. BIOH and CKH are healthy samples collected in bioorganic fertilizer (BIO) and control (CK) treatments, respectively. BIOD and CKD are diseased samples collected in BIO and CK, respectively

Supplementary tables

Table S1 Similarity percentage analysis showing the top 20 contributing bacterial genera to the difference between disease suppressive (CKH) and diseased (CKD) samples in the control treatment

Indicator Genus	CKD (%)	CKH (%)	*SumCum (%)
Bacillus	0.7 ± 0.6	**7.6 ± 4.5**	5.5
Acidobacteria-Gp6	**4.2 ± 1.3**	2.9 ± 2.3	10.2
Gemmatimonas	**6.0 ± 1.6**	2.8 ± 1.1	13.8
Acidobacteria-Gp1	0.2 ± 0.1	**1.1 ± 1.2**	19.4
<u>***Ohtaekwangia***</u>	**3.3 ± 1.2**	0.7 ± 0.7	23.8
Steroidobacter	**2.3 ± 2.9**	0.5 ± 0.3	27.4
Burkholderia	NA	**0.1 ± 0.1**	28.9
Streptomyces	0.2 ± 0.2	**2.1 ± 1.6**	30.4
Streptophyta	**1.5 ± 2.9**	NA	32.8
Acidobacteria-Gp4	**0.7 ± 0.3**	0.7 ± 0.6	34.0
Sphingomonas	0.3 ± 0.2	**0.4 ± 0.2**	35.1
Denitratisoma	**0.1 ± 0.1**	NA	36.2
Dongia	**1.6 ± 0.6**	0.8 ± 0.1	37.2
Actinophytocola	NA	**1.1 ± 2.1**	39.2
Acidobacteria-Gp16	0.7 ± 0.6	**1.1 ± 0.8**	40.1
Tumebacillus	0.2 ± 0.2	**1.1 ± 0.7**	40.9
Terrimonas	**0.3 ± 0.2**	0.2 ± 0.2	41.6
Nocardioides	0.6 ± 0.9	**0.7 ± 0.5**	42.4
Spartobacteria	0.1 ± 0.2	**0.3 ± 0.2**	43.2
Bradyrhizobium	0.2 ± 0.1	**0.5 ± 0.3**	43.9

*Cumulative dissimilarity, based on Bray-Curtis distance.

Indicator genus name in bold indicates a significant difference (t-test, $p < 0.05$).

Underlined indicator genus indicates a significant difference (t-test, $p < 0.01$).

Numbers in bold represent relatively higher mean value in comparison.

NA is less than 0.005 or not detected.

Table S2 Similarity percentage analysis showing the top 20 contributing bacterial genera to the difference between diseased samples (CKD) in control treatment and healthy samples (BIOH) in the bioorganic fertilizer treatment

Indicator Genus	BIOH (%)	CKD (%)	*SumCum (%)
Acidobacteria-Gp6	**6.5 ± 4.0**	4.2 ± 1.3	6.1
Gemmatimonas	4.2 ± 2.2	**6.0 ± 1.6**	10.5
Ohtaekwangia	1.0 ± 0.5	**3.3 ± 1.2**	14.4
<u>***Acidobacteria*-Gp1**</u>	**2.0 ± 0.9**	0.2 ± 0.1	17.8
Steroidobacter	1.1 ± 0.1	**2.3 ± 2.9**	20.7
Bacillus	**2.2 ± 1.1**	0.7 ± 0.6	23.3
Streptophyta	0.1 ± 0.1	**1.5 ± 2.9**	25.9
Flavobacterium	**1.2 ± 0.8**	NA	28.1
<u>***Dongia***</u>	0.4 ± 0.1	**1.6 ± 0.6**	30.1
<u>***Acidobacteria*-Gp4**</u>	**1.8 ± 0.5**	0.7 ± 0.3	32.1
Sphingobium	**1.0 ± 1.4**	NA	33.9
<u>***Spartobacteria***</u>	**0.9 ± 0.3**	0.1 ± 0.2	35.4
Burkholderia	**0.8 ± 0.5**	NA	36.7
<u>***Hyphomicrobium***</u>	0.1 ± NA	**0.8 ± 0.4**	37.9
Haliscomenobacter	**0.7 ± 0.8**	0.4 ± 0.2	39.0
Massilia	**0.6 ± 0.4**	NA	40.1
<u>***Pseudomonas***</u>	**0.7 ± 0.2**	0.1 ± 0.1	41.2
Sorangium	0.3 ± 0.1	**0.7 ± 0.7**	42.2
Cupriavidus	**0.6 ± 0.8**	0.1 ± 0.1	43.2
Flavisolibacter	**0.5 ± 0.4**	NA	44.1

*Sum cumulative dissimilarity based on Bray-Curtis distance.

Indicator genus name in bold indicates a significant difference (t-test, $p < 0.05$).

Underlined indicator genus indicates a significant difference (t-test, $p < 0.01$).

Numbers in bold represent relatively higher mean value in comparison.

NA is less than 0.005 or not detected.
